# A pan-cancer analysis of copper homeostasis-related gene lipoyltransferase 1: Its potential biological functions and prognosis values

**DOI:** 10.3389/fgene.2022.1038174

**Published:** 2022-10-18

**Authors:** Ying Liu, Gengqiu Luo, Yuanliang Yan, Jinwu Peng

**Affiliations:** ^1^ Department of Pathology, Xiangya Changde Hospital, Changde, China; ^2^ Department of Pathology, Xiangya Hospital, Basic School of Medicine, Central South University, Changsha, China; ^3^ National Clinical Research Center for Geriatric Disorders, Xiangya Hospital, Central South University, Changsha, China; ^4^ Department of Pharmacy, Xiangya Hospital, Central South University, Changsha, China

**Keywords:** LIPT1, pan-cancer, biological functions, prognosis, immune

## Abstract

As a key copper homeostasis-related molecule, lipoyltransferase 1 (LIPT1) is an essential enzyme for the activation of mitochondrial 2-ketoacid dehydrogenase, participating in fatty acylation. However, the biological significances of LIPT1 in the pan-cancer are unclear. Here, we comprehensively analyzed the functional characteristics of LIPT1 in human cancers and its roles in immune response. We found that LIPT1 was down-regulated in some cancers. And LIPT1 overexpression is associated with favorable prognosis in these patients, such as breast cancer, clear cell renal cell carcinoma, ovarian cancer and gastric cancer. We also explored the mutational status and methylation levels of LIPT1 in human cancers. Gene enrichment analysis indicated that abnormally expressed LIPT1 was significantly associated with immune cells infiltration, such as B cells, CD8^+^ T cells and cancer-associated fibroblast cells. The result from single cell sequencing reflected the important roles of LIPT1 in the regulation of several biological behaviors of cancer cells, such as DNA damage response and cell apoptosis. Taken together, our research could provide a comprehensive overview about the significances of LIPT1 in human pan-cancer progression, prognosis and immune.

## Introduction

Cancers are global health problems, affecting human health and quality of life. According to the statistics from World Health Organization, cancers are the main endangering cause of human life (Tao et al., 2021; Yu and Mitrofanova, 2021). Identifying the valuable pan-cancer genes would be crucial to clarify the underlying mechanisms for occurrence and development of different tumors (He et al., 2021; Shi et al., 2021).

Lipoic acid (LA), an eight-carbon fatty acid, serves as an important cofactor for the mitochondrial glycine cleavage system (Habarou et al., 2017). Lipoyltransferase 1 (LIPT1), as a lipoate-specific sequential enzymes, could be used to maintain the oxidative and reductive glutamine metabolism (Ni et al., 2019). Recent studies have established the functional link between aberrant LIPT1expression and tumorigenesis, including bladder cancer (Chen et al., 2021) and melanoma (Lv et al., 2022). In melanoma patients, Lv and colleague found that upregulated LIPT1 expression might suppress the infiltration of regulatory T cells (Tregs), thereby enhancing the immunotherapy efficacy (Lv et al., 2022). However, the detailed roles of LIPT1 in different tumor types remains elusive.

In this study, by using multiple bioinformatics methods, we explored the underlying molecular mechanisms of LIPT1 in the pathogenesis and clinical prognosis of multiple human cancers. The expression profiles of LIPT1 and corresponding survival status were comprehensively analyzed using the datasets from TCGA and GEO. Meanwhile, gene enrichment indicated the roles of LIPT1-related molecules in tumorigenesis. Meanwhile, the potential implications of LIPT1 in anti-tumor immune response were also explored.

## Materials and methods

### Gene expression analysis

TIMER2.0 (Li et al., 2020) was used to investigate the different expression profiles of LIPT1 between pan-cancer and adjacent normal tissues. The gene expression levels were shown using a log2 (TPM + 1) scale, where TPM stands for transcripts per million. The clinical proteomic tumor analysis consortium (CPTAC) (Edwards et al., 2015) in UALCAN database (Chandrashekar et al., 2022) was used to analyze the protein expression of LIPT1 in pan-cancers. Gene Expression Profiling Interactive Analysis 2 (GEPIA2.0) (Tang et al., 2017) was used to analyze the relationship between LIPT1 expression and patients’ pathological stage in all TCGA cancers. The Human Protein Atlas (HPA) (Colwill et al., 2011) was further used to confirm the intensity of LIPT1 immunohistochemical staining in several cancer tissues, including kidney cancer, breast carcinoma, and uterus endometrium adenocarcinoma.

### Survival analysis

The expression of LIPT1 on the patients’ prognostic values, including overall survival (OS) and disease-free survival (DFS), was obtained from GEPIA2.0. TCGA tumor patients were divided into the high-expression and low-expression cohorts based on the cut-off values (50% and 50%). The hazards ratio was calculated based on Cox PH Model. With the log-rank test, Kaplan-Meier plotter (Lanczky and Gyorffy, 2021) was used to perform the survival analysis in tumors.

### Genetic alteration analysis

cBioPortal (Gao et al., 2013) was used to collect the alteration frequency, mutation type, mutation site information, and three-dimensional (3D) structure of candidate proteins in all TCGA tumors. In the “Comparison” module, clinical prognosis data, including progression-free survival (PFS), disease-specific survival (DSS), DFS, and OS, for all TCGA cancer types with or without LIPT1 gene alterations were downloaded and analyzed.

### The infiltration of immune cells

Using multiple algorithms, such as TIMER, EPIC, MCPCOUNTER, XCELL and TIDE, we applied TIMER2.0 tool to evaluate the correlation of LIPT1 expression with immune infiltration levels in different TCGA cancers.

### Single cell sequencing

CancerSEA (Yuan et al., 2019) is a specialized single cell sequencing database, which can provide different functional status of cancer cells at the single cell level. The correlation data between LIPT1 expression and different tumor function based on single cell sequencing data were analyzed. T-SNE diagrams demonstrated the expression profiles of LIPT1 at single cells in TCGA samples.

### Gene enrichment analysis

BioGRID website (Oughtred et al., 2021) was used to analysis the protein-protein interaction network. GEPIA2.0 was used to obtain the top 100 LIPT1-correlated genes from all TCGA tumor and normal tissues. Then we conducted a pairwise gene-gene Pearson correlation analysis between LIPT1 and the selected genes. Gene ontology (GO) and Kyoto encyclopedia of genes and genome (KEGG) enrichment analyses were used to investigate the underlying biological functions and signaling pathways affected by LIPT1 in TCGA tumors. *p*-value < 0.05 was considered to be statistically significant.

## Results

### The different expression profiles of lipoyltransferase 1 in human pan-cancer

**FIGURE 1:**
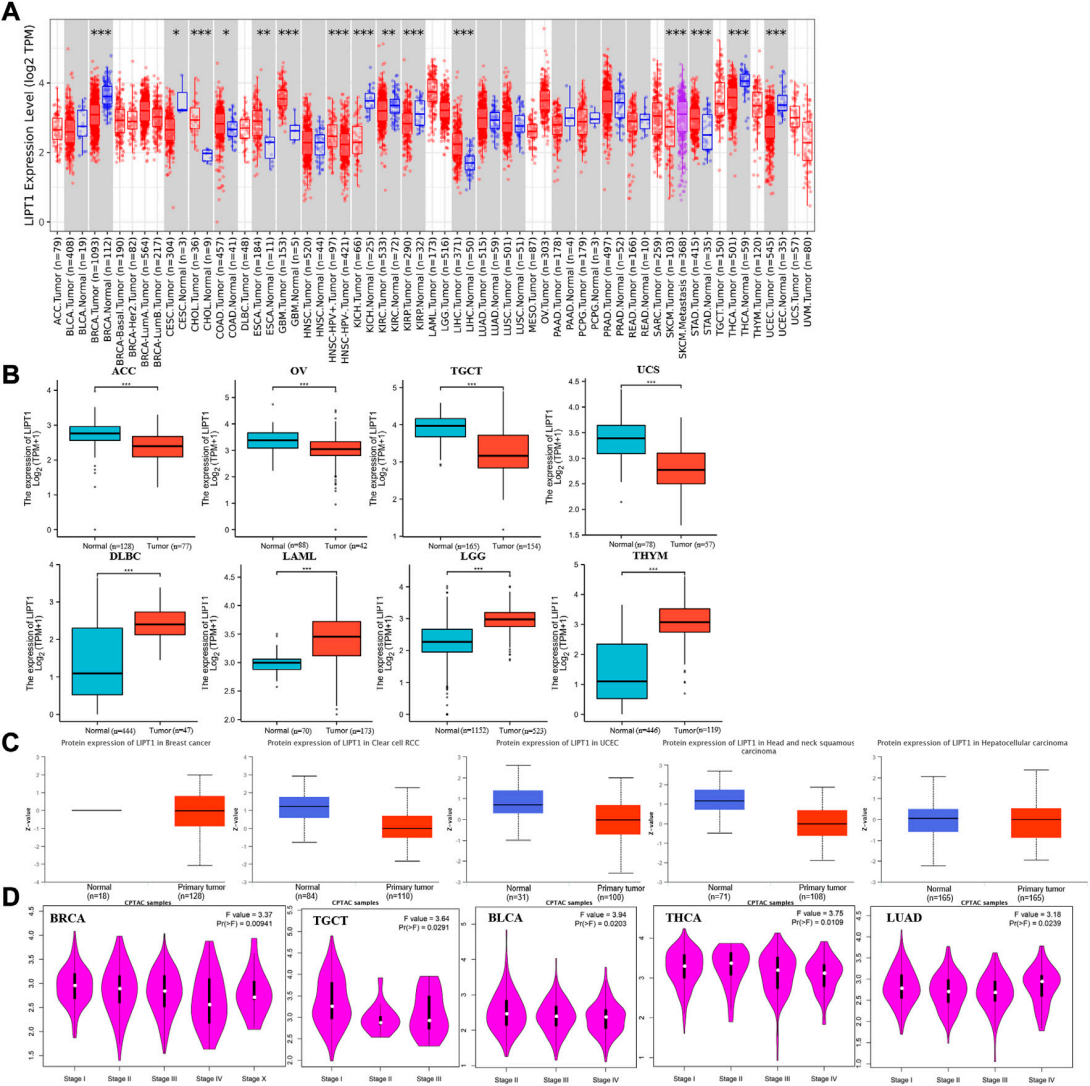
The expression of LIPT1 in pan-cancer. **(A)** LIPT1 expression in different cancers from TIMER2.0. *p < 0.05; **p < 0.01; ***p < 0.001. **(B)** The expression differences of LIPT1 in ACC, OV, TGCT, UCS, DLBC, LAML, LGG, and HNSC from GTEx and TCGA. ***p < 0.001. **(C)** The protein levels of LIPT1 in BRCA, KIRC, UCEC, HNSC, and LIHC were analyzed using CPTAC. **(D)** LIPT1 expression levels and the pathological stages were analyzed using GEPIA2.0.

Initially, we examined LIPT1 expression levels in pan-cancer by TIMER2.0. As shown in Figure 1A, the analysis revealed that LIPT1 expression was significantly lower in various tumors than in the adjacent normal tissues, including breast invasive carcinoma (BRCA), cervical squamous cell carcinoma and endocervical adenocarcinoma (CESC), kidney renal clear cell carcinoma (KIRC), kidney renal papillary cell carcinoma (KIRP), thyroid carcinoma (THCA), uterine corpus endometrial carcinoma (UCEC), kidney chromophobe (KICH). Conversely, LIPT1 expression was significantly up-regulated in some other tumors, including cholangiocarcinoma (CHOL), colon adenocarcinoma (COAD), esophageal carcinoma (ESCA), glioblastoma multiforme (GBM), liver hepatocellular carcinoma (LIHC) and stomach adenocarcinoma (STAD). However, no significantly differential expression of LIPT could be found in other tumors, such as bladder urothelial carcinoma (BLCA), head and neck squamous cell carcinoma (HNSC), and so on. Given some data in the normal tissues were not available, we further examined the expression differences of LIPT1 using the TCGA and GTEx datasets. As shown in Figure 1B, we found the down-regulated LIPT1 in adrenocortical carcinoma (ACC), ovarian serous cystadenocarcinoma (OV), testicular germ cell tumors (TGCT) and uterine carcinosarcoma (UCS). Meanwhile, we found the up-regulated LIPT1 in lymphoid neoplasm diffuse large B-cell lymphoma (DLBC), acute myeloid leukemia (LAML), brain lower grade glioma (LGG) and thymoma (THYM).

Then, the National Cancer Institute’s CPTAC dataset was used to assessed LIPT1 expression at a protein level. We found that the total protein expression of LIPT1 was significantly down-regulated in BRCA, KIRC, UCEC, HNSC, and LIHC (Figure 1C). We also used GEPIA2.0 to explore the correlation between LIPT1 expression level and the pathological stages of tumors. And we found the obvious effect of LIPT1 expression on the patients’ stages in BRCA, TGCT, BLCA, THCA and lung adenocarcinoma (LUAD) (Figure 1D; Supplementary Figure S1).

**FIGURE 2:**
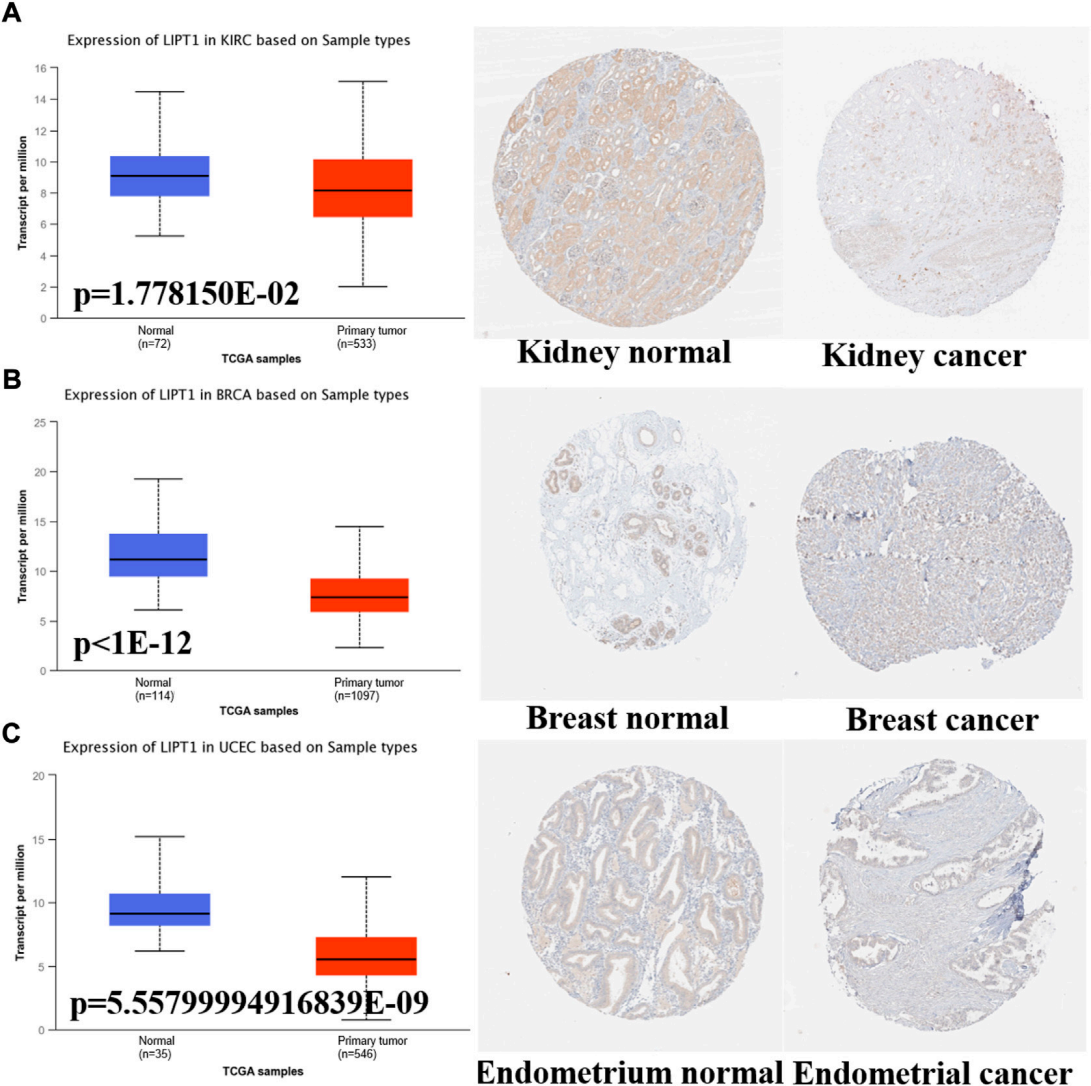
The different expression of LIPT1 between normal tissues and tumor tissues. **(A–C)** UALCAN and HPA platforms displayed the downregulated expression of LIPT1 in tumor tissue derived from kidney, breast and endometrium.

Meanwhile, we further confirmed the LIPT1 expression using the IHC results provided by the HPA database. The IHC staining of LIPT1 was mainly weakly or negatively expressed in tumor tissue derived from kidney cancer, breast cancer and endometrial cancer (Figures 2A–C). Overall, we demonstrated the decreased LIPT1 expression in these tumors.

### The prognostic values of lipoyltransferase 1 on the patients’ survival

**FIGURE 3:**
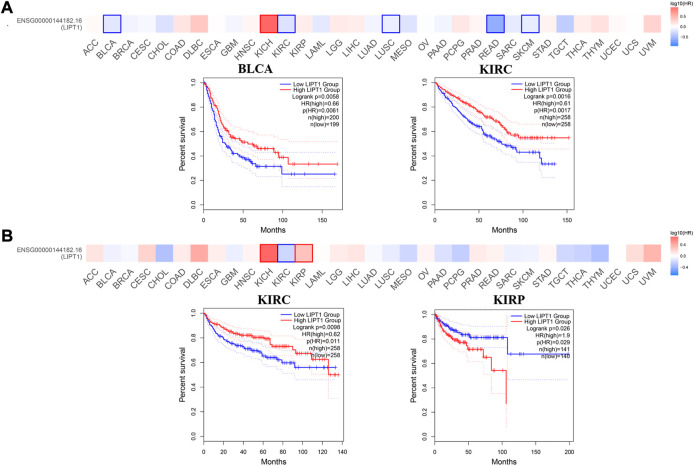
Prognostic values of LIPT1 expression in pan-cancer. **(A,B)** GEPIA2.0 was used to analyze the effects of LIPT1 gene expression on the patients’ prognosis in pan-cancer, including OS **(A)** and DFS **(B)**.

Next, GEPIA2.0 database was used to evaluate the values of LIPT1 on patients’ prognosis, including OS and DFS. We found that higher LIPT1 expression was significantly associated with increased OS in BLCA (*p* = 0.0061) and KIRC (*p* = 0.0017) (Figure 3A). And DFS analysis data showed that high expression LIPT1 was associated with favorable prognosis in KIRC (*p* = 0.011). In contrast, in KIRP patients, high expression of LIPT1 is associated with poor prognosis (*p* = 0.029) (Figure 3B). Meanwhile, we also used the Kaplan-Meier plotter tool to analyze the survival data. Correspondingly, in breast cancer, high expression of LIPT1 was related to good PFS (*p* = 1.9e-06), OS (*p* = 3.1e-06) and distant metastasis-free survival (DMFS) (p = 2e-07). In addition, high LIPT1 expression was significantly associated with increased DMFS in ovarian cancer (*p* = 0.013) and decreased post progression survival (PPS) in gastric cancer (*p* = 0.00077) (Supplementary Figures S2A–C). Therefore, these results demonstrated that LIPT1 may be a potential prognostic marker in various cancers. Especially, the expression profiles and prognostic values indicated that LIPT1 might act as a tumor suppressor gene in breast cancer patients.

### Lipoyltransferase 1 mutation in various tumors

**FIGURE 4:**
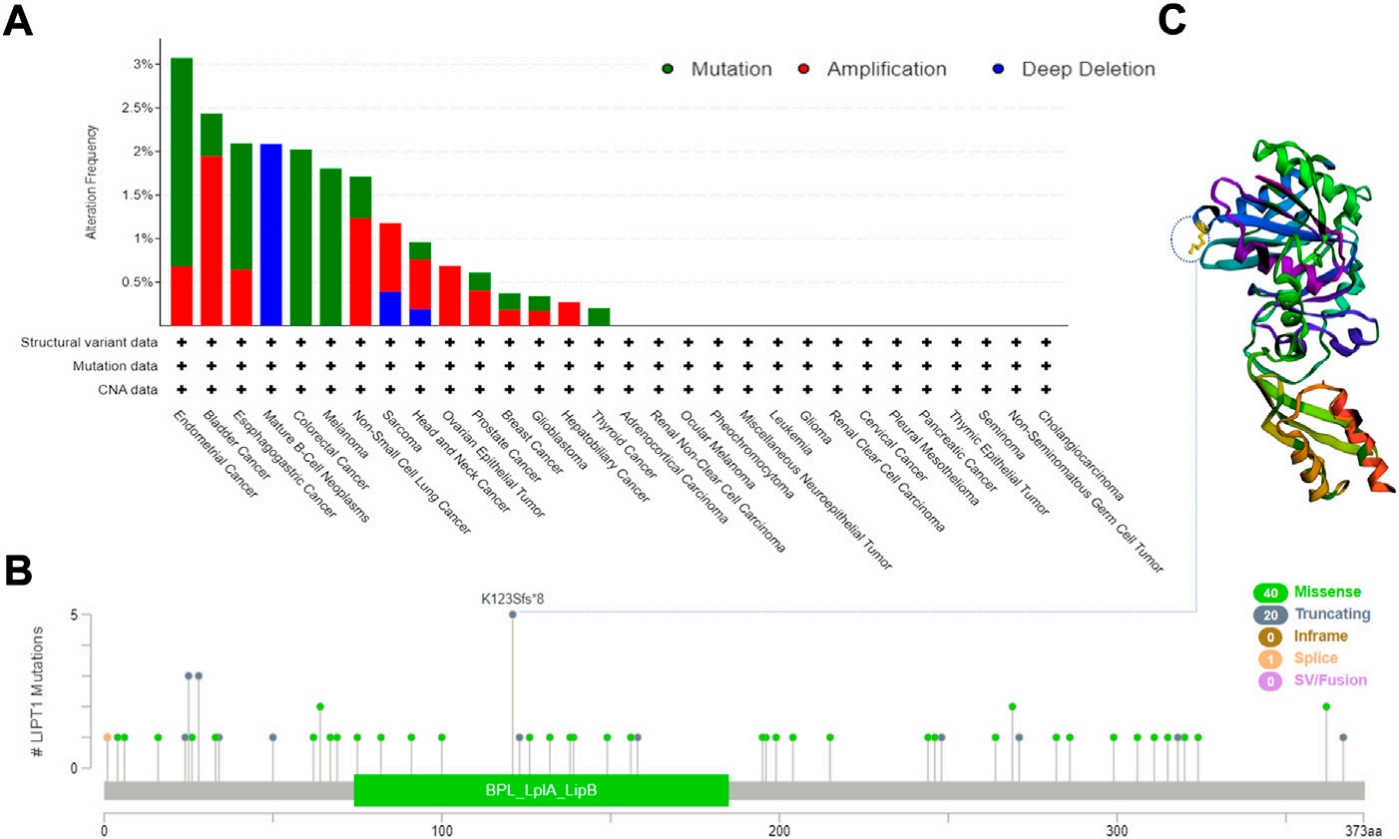
LIPT1 gene mutation in various cancers. **(A,B)** cBioPortal was used to display the alteration frequency of different mutation types **(A)** and mutation site **(B)** of LIPT1 in pan-cancer. **(C)** K123sf*8 mutation site was shown in the 3D protein structure of LIPT1.

To explore the gene mutation of LIPT1 in various cancers, we analyzed its mutation status through cBioPortal platform based on TCGA data. Pan-cancer analysis suggested the high LIPT1 amplification in BLCA (>2%) and high mutation in UECE (>3%). Mature B cell neoplasms had the highest incidence of “deep deletion” with the frequency of ∼2% (Figure 4A). As shown in Figure 4B, we found that missense and truncating were the predominant mutation styles in LIPT1. For instance, a truncating mutation within the BPL_LplA_LipB domain, K123sf*8 alteration, could be detected in five STAD cases. Figure 4C displayed the K123sf*8 alteration in the 3D structure of LIPT1 protein. In addition, we analyzed the potential links between genetic alterations of LIPT1 and the survival prognosis of patients in pan-cancers. However, we could not find the obvious effect of LIPT1 genetic alterations on the patients’ prognosis (Supplementary Figures S3A–F, S4A–F). These unexpected results need to be further verified with more clinical patient data.

### Promoter methylation of lipoyltransferase 1 in human cancers

**FIGURE 5:**
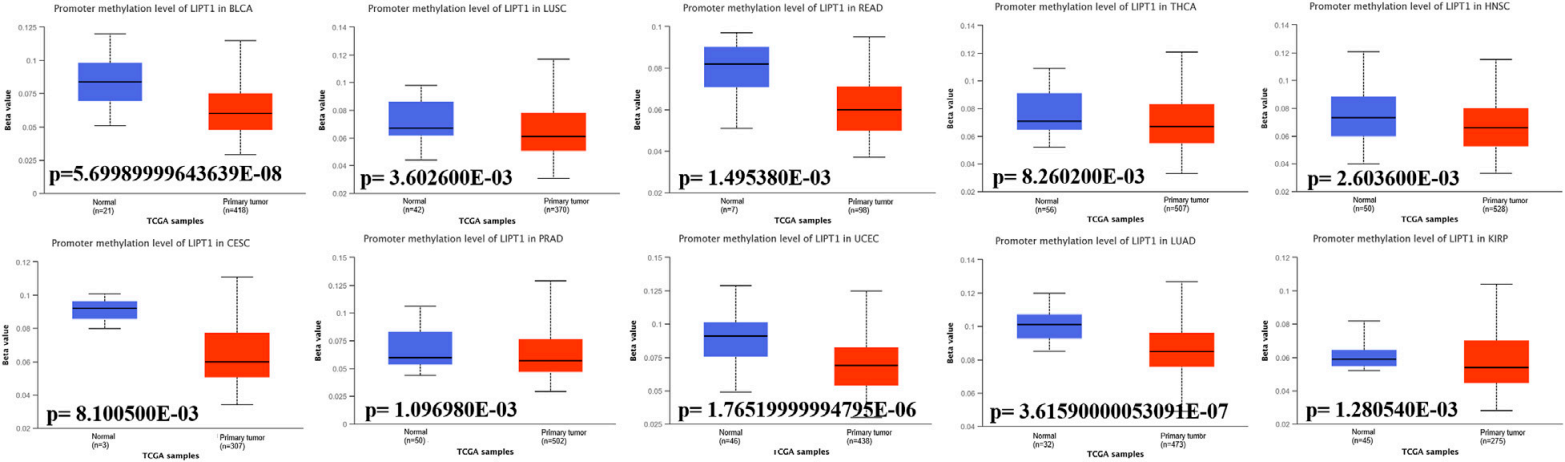
Promoter methylation levels of LIPT1 in cancers. The methylation values of LIPT1 between normal and primary tumor tissues were analyzed using UALCAN tool.

Promoter DNA methylation has been proved to affect the transcriptional repression and participate in the tumor oncogenesis (Smith et al., 2020). We compared the methylation values of LIPT1 between normal and tumor tissues. Our analysis results displayed that promoter methylation levels of LIPT1 were significantly reduced in several tumor tissues, including BLCA, lung squamous cell carcinoma (LUSC), rectum adenocarcinoma (READ), THCA, HNSC, CESC, prostate adenocarcinoma (PRAD), UCEC, LUAD, and KIRP (Figure 5). In contrast, the promoter methylation levels of LIPT1were not significantly different in other tumors (Supplementary Figure S5). These results suggested that the transcriptional expression of LIPT1 may be due to the alterations of promoter methylation.

### The roles of lipoyltransferase 1 on the regulation of immune cell infiltration

**FIGURE 6:**
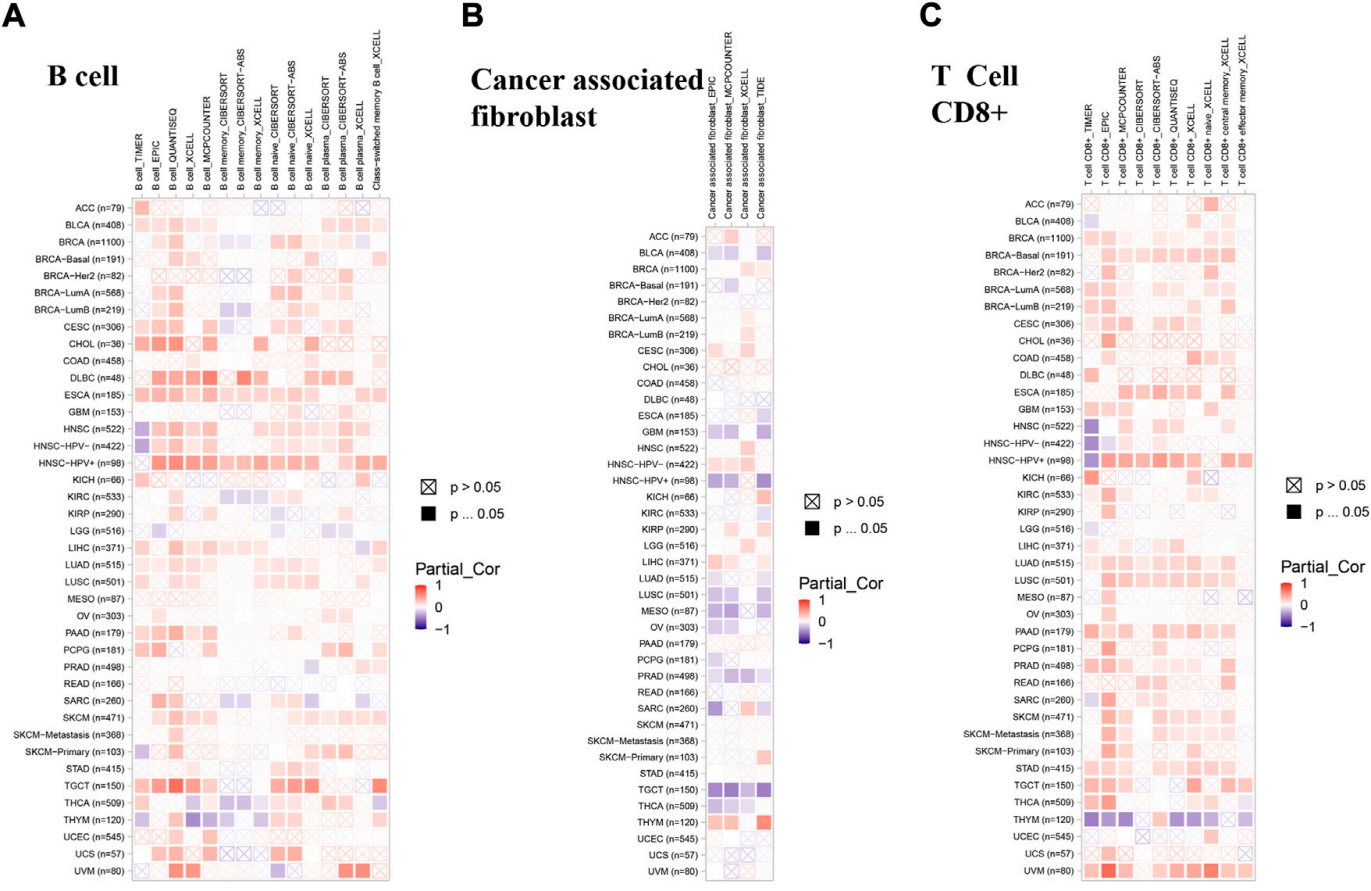
The correlation between immune cells and LIPT1 expression in cancers. **(A–C)** The relationship between LIPT1 expression and immune infiltration of B cell **(A)**, cancer-associated fibroblast **(B)** and T cell CD8^+^ **(C)** was depicted by TIMER2.0 database. Several algorithms, such as TIMER, EPIC, QUANTISEQ, XCELL, MCPCOUNTER, CIBERSORT, CIBERSORT-ABS, and TIDE, were applied to explore the correlation. Positive correlation (0–1) are indicated with the red color, while negative correlation (−1 to 0) are indicated with the blue color. *p*-value < 0.05 is considered as statistically significant. A cross indicates non-significant correlations.

Recent researches have shown that immune infiltration is associated with the initiation, progression, and metastasis in human cancers (Stenstrom et al., 2021; Ren et al., 2022; Shan et al., 2022). Several algorithms, such as TIMER, EPIC, QUANTISEQ, XCELL, MCPCOUNTER, CIBERSORT, CIBERSORT-ABS, and TIDE, were applied to explore the correlation between LIPT1 expression and the infiltration of different immune cells in pan-cancer. We found a positive correlation between the infiltration of B cells and LIPT1 expression in BLCA, ESCA, pancreatic adenocarcinoma (PAAD) and TGCT (Figure 6A). Meanwhile, a negative correlation between the infiltration of cancer-associated fibroblasts and LIPT1 expression could be found in PRAD and TGCT (Figure 6B). In LUAD, LIPT1 expression was positively correlated with CD8^+^ T cells infiltration (Figure 6C). We found no significantly correlation between LIPT1 expression and the infiltration values of dendritic cells (DC), monocyte, regulatory T cells (Treg), natural killer cells (NK), neutrophil and macrophage (Supplementary Figure S6). These findings demonstrated that LIPT1 may act as a novel immune-associated biomarkers for tumor development.

### The expression pattern of lipoyltransferase 1 at single-cell levels

**FIGURE 7:**
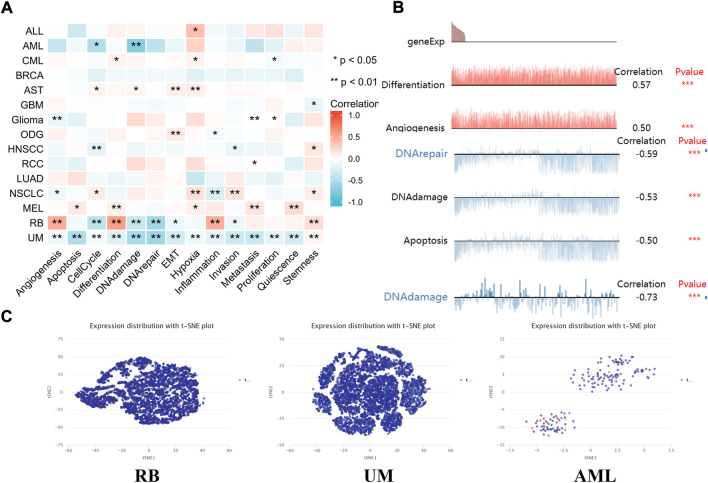
The expression levels of LIPT1 at single-cell levels. **(A,B)** The relationship between LIPT1 expression and different functional states in tumors was explored by the CancerSEA tool. *p < 0.05; **p < 0.01; ***p < 0.001. **(C)** LIPT1 expression profiles were shown at single cells from RB, UM and AML by T-SNE diagram.

Single-cell transcriptome sequencing is a key technique for analyzing the underlying functions of candidate molecules at single-cell levels (He et al., 2021; Li et al., 2021). In retinoblastoma (RB), the expression of LIPT1 was negatively associated with cell cycle, DNA repair response, EMT and invasion. By contrast, LIPT1 expression was positively related to angiogenesis, differentiation, inflammation and stemness. LIPT1 expression in uveal melanoma (UM) had a negative relationship with almost all tumor biological behaviors, such as cell death, DNA damage response, invasion and metastasis. In addition, the results demonstrated that LIPT1 expression was negatively related with cell cycle and DNA damage in acute myelocytic leukemia (AML) (Figure 7A). In addition, Figure 7B displayed the significant correlation between the LIPT1 expression and differentiation in RB, angiogenesis, DNA damage, DNA repair, and apoptosis in UM, and DNA damage in AML. Moreover, LIPT1 expression profiles were shown at single cell levels from RB, UM, and AML by T-SNE diagram (Figure 7C).

### Functional enrichment analysis of lipoyltransferase 1-related genes in cancers

**FIGURE 8:**
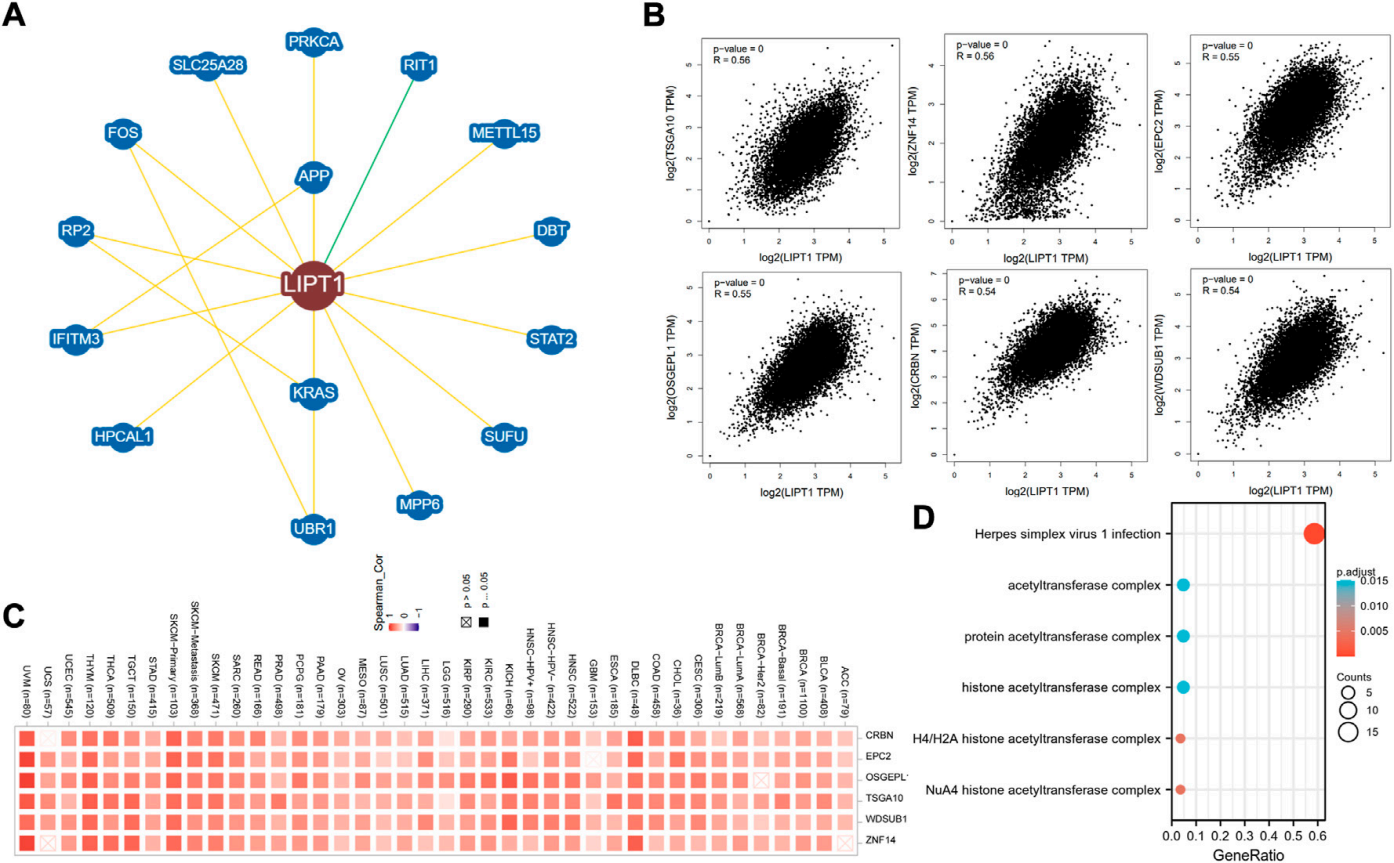
Functional enrichment analysis of LIPT1-related genes. **(A)** LIPT1-related genes were obtained from the BioGRID web tool, and 15 proteins were displayed. **(B)** GEPIA2.0 showed the positive correlations between LIPT1 and six genes (TSGA10, ZNF14, EPC2, OSGEPL1, CRBN, and WDSUB1). *p*-value < 0.001. **(C)** The heatmap confirmed that LIPT1 expression was positively correlated with the six genes (TSGA10, ZNF14, EPC2, OSGEPL1, CRBN, and WDSUB1) in pan-cancer. **(D)** GO and KEGG enrichment analyses of LIPT1-related genes.

Next, we used functional enrichment analysis to evaluate the underlying molecular mechanisms of LIPT1 in tumorigenesis and development. As shown in Figure 8A, the 15 interacting molecules with LIPT1 were obtained from BioGRID web tool. In addition, we acquired the top 100 LIPT1 co-expressed genes (Supplementary Table S1) in pan-cancer from GEPIA2.0. Among these, testis specific 10 (TSGA10), zinc finger protein 14 (ZNF14), enhancer of polycomb homolog 2 (EPC2), o-sialoglycoprotein endopeptidase like 1 (OSGEPL1), cereblon (CRBN) and wd repeat, sterile alpha motif and u-box domain containing 1 (WDSUB1) showed high correlations with LIPT1 in the majority of cancer types (Figures 8B,C). Meanwhile, GO and KEGG enrichment analyses in Figure 8D indicated that the roles of LIPT1 co-expressed genes on the regulation of herpes simplex virus one infection and acetyltransferase complex in tumorigenesis and development.

## Discussion

Emerging studies have shown that copper death plays an important role in the occurrence and treatment of human tumors (Jiang et al., 2022). In our research, we performed a comprehensive analysis of LIPT1, the copper death-related gene, in a total of 33 different tumors. The expression of LIPT1 was significantly decreased in several tumor tissues, including BRCA, CESC, KIRC, KIRP, THCA, UCEC, and KICH. Overexpression of LIPT1 is associated with favorable prognosis in tumor patients, such as breast cancer, clear cell renal cell carcinoma, ovarian cancer, and gastric cancer. In addition, abnormally expressed LIPT1 was significantly associated with immune cells infiltration, such as B cells, CD8^+^ T cells, and cancer associated fibroblast cells. Therefore, LIPT1 might be a potential prognosis biomarker and immune target for tumor patients.

Copper is a co-factor for important enzymes in all organisms (Kahlson and Dixon, 2022). Unbalanced copper homeostasis affects tumor cell growth, causing irreversible damage (Jiang et al., 2022). Studies showed that copper homeostasis might be regulated by protein lipoylation (Tsvetkov et al., 2022), protein misfolding (Gupta et al., 2022) and DNA damage response (He et al., 2020). Abnormal accumulation of intracellular copper induces a new mode of cell death, copper drooping (Kahlson and Dixon, 2022). The copper homeostasis has been associated with the development and prognosis of patients with various tumors (Feng et al., 2022; Huang et al., 2022; Lei et al., 2022; Li et al., 2022; Wang et al., 2022). As a copper death-related gene, LIPT1 is required for lipoylation and activation of 2-ketoacid dehydrogenases in humans (Tort et al., 2014). LIPT1 genetic alterations, including mutation and deep deletion, cause a variety of human diseases, such as Leigh disease (Soreze et al., 2013) and nonketotic hyperglycinemia with early-onset convulsions (Mayr et al., 2014). However, few studies have established the functional link between LIPT1 and tumorigenesis. Even though LIPT1 has been proved to be upregulated in melanoma (Chen et al., 2021), the detailed roles and underlying mechanisms of LIPT1 in human cancers are unclear and warrant further exploration. Our exploratory findings demonstrated that LIPT1 genetic alterations, including mutation and deep deletion, could be observed in a variety of cancers. At the same time, there were significant differences in LIPT1 methylation levels between tumor tissues and normal tissues. And single cell sequencing and gene enrichment indicated that LIPT1-correlated gene might regulate several cancer biological functions, such as DNA damage response and cell death.

The infiltrating immune cells play essential roles in regulating cancer cell recognition and tumor growth (Shihab et al., 2020; Talty and Olino, 2021). The most well-known function of B cells is to produce antibodies, such as IgM, IgG, IgE, and IgA (Kim et al., 2021). The depleted effector B and T cells could help tumor cells to evade immune surveillance, thereby reducing overall survival in tumor patients (Chakraborty et al., 2022). In this study, we found that LIPT1 expression was strongly correlated with the infiltration of immune cells, including B cell, cancer-associated fibroblast and CD8^+^ T cells. These results suggested that LIPT1 could be an effective target for immunotherapy, and provided new hope for clinical treatment of tumor patients. The association between LIPT1 expression and immune checkpoints in cancer patients still needs to be explored in more preclinical and clinical trials.

In conclusion, using comprehensive bioinformatics analysis techniques, we explored the expression levels, clinical prognosis, methylation values, genetic alterations, and immunomodulatory effects of LIPT1 in pan-cancer. The results suggested that LIPT1 may be a novel potential prognostic and immune-associated biomarker for cancer patients. This study lays the foundation for further research on the specific mechanisms of LIPT1 in the development and treatment of different tumors.

## Data Availability

The datasets presented in this study can be found in online repositories. The names of the repository/repositories and accession number(s) can be found in the article/Supplementary Material.

## Supplementary material

SUPPLEMENTARY FIGURE S1 LIPT1 expression levels and the pathological stages of tumors was analyzed using GEPIA2.0.

SUPPLEMENTARY FIGURE S2
**(A-C)** Kaplan-Meier plotter tool was used to analyze the effects of LIPT1 expression on the patients’ prognosis from breast cancer, ovarian cancer and gastric cancer.

SUPPLEMENTARY FIGURE S3
**(A-F)** cBioPortal tool was used to explore the effects of LIPT1 mutation on the prognosis of patients from KIRC, SKCM, BLCA, GBM, UCEC, and BRCA, including OS, DSS, DFS, and PFS.

SUPPLEMENTARY FIGURE S4
**(A-F)** cBioPortal tool was used to explore the effects of LIPT1 mutation on the prognosis of patients from LIHC, stomach and esophageal carcinoma (STES), COAD, HNSC, THCA, and OV, including OS, DSS, DFS, and PFS.

SUPPLEMENTARY FIGURE S5 The methylation values of LIPT1 between normal and primary tumor tissues were analyzed by UALCAN dataset.

SUPPLEMENTARY FIGURE S6 The relationship between LIPT1 expression and immune infiltration of DC, monocyte, Treg, NK, neutrophil, macrophage was depicted by TIMER2.0 database.

SUPPLEMENTARY TABLE S1 The top 100 LIPT1 co-expressed genes in pan-cancer from GEPIA2.0.
