# Supplementary figures and images for: A pan-cancer analysis of copper homeostasis-related gene lipoyltransferase 1: Its potential biological functions and prognosis values
Source: Front Genet. 2022 Oct 18;13:1038174. doi: 10.3389/fgene.2022.1038174 (PMC9623413; doi:10.3389/fgene.2022.1038174)

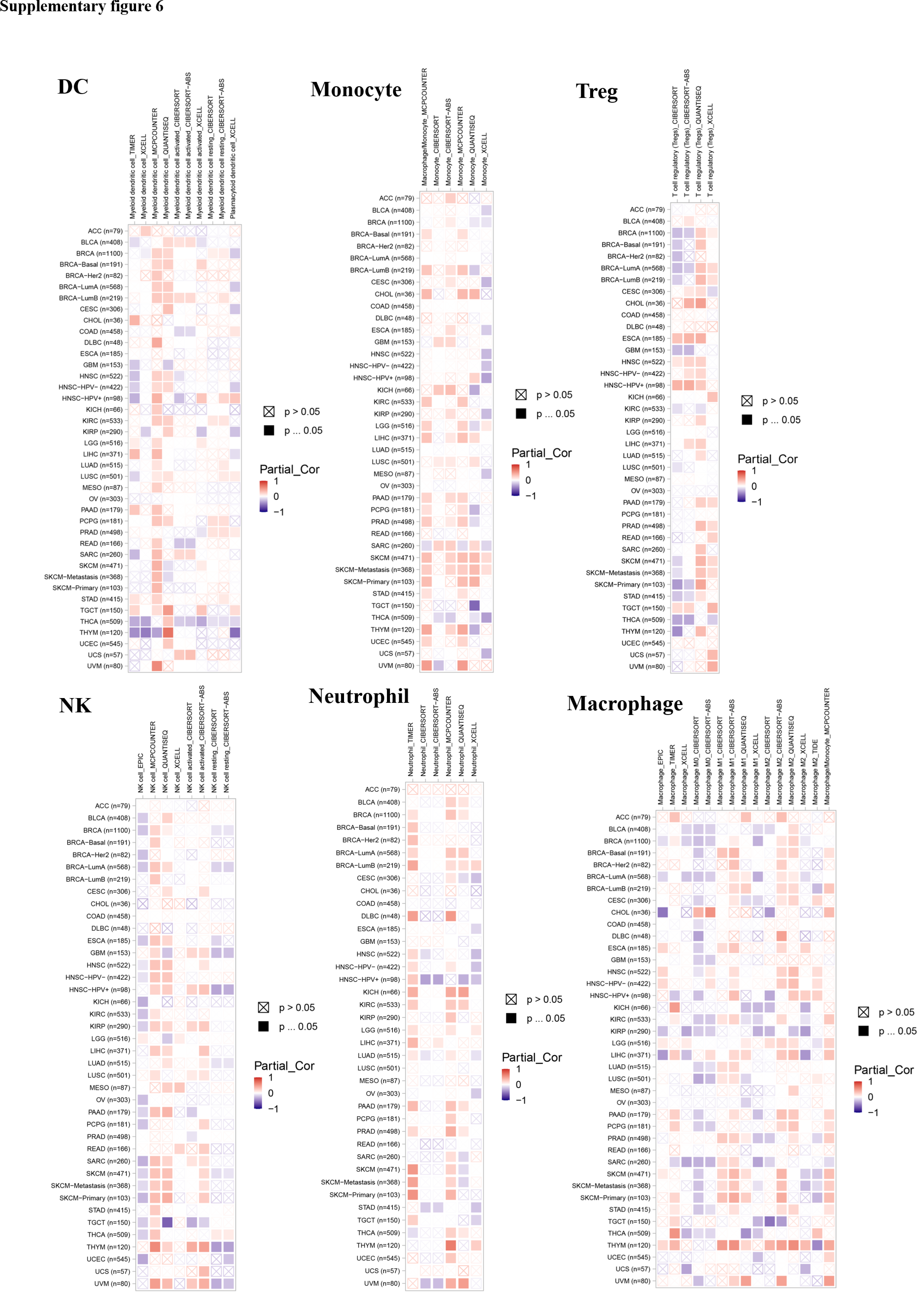

Supplement: Supplementary file 1 [file Image6.TIF]

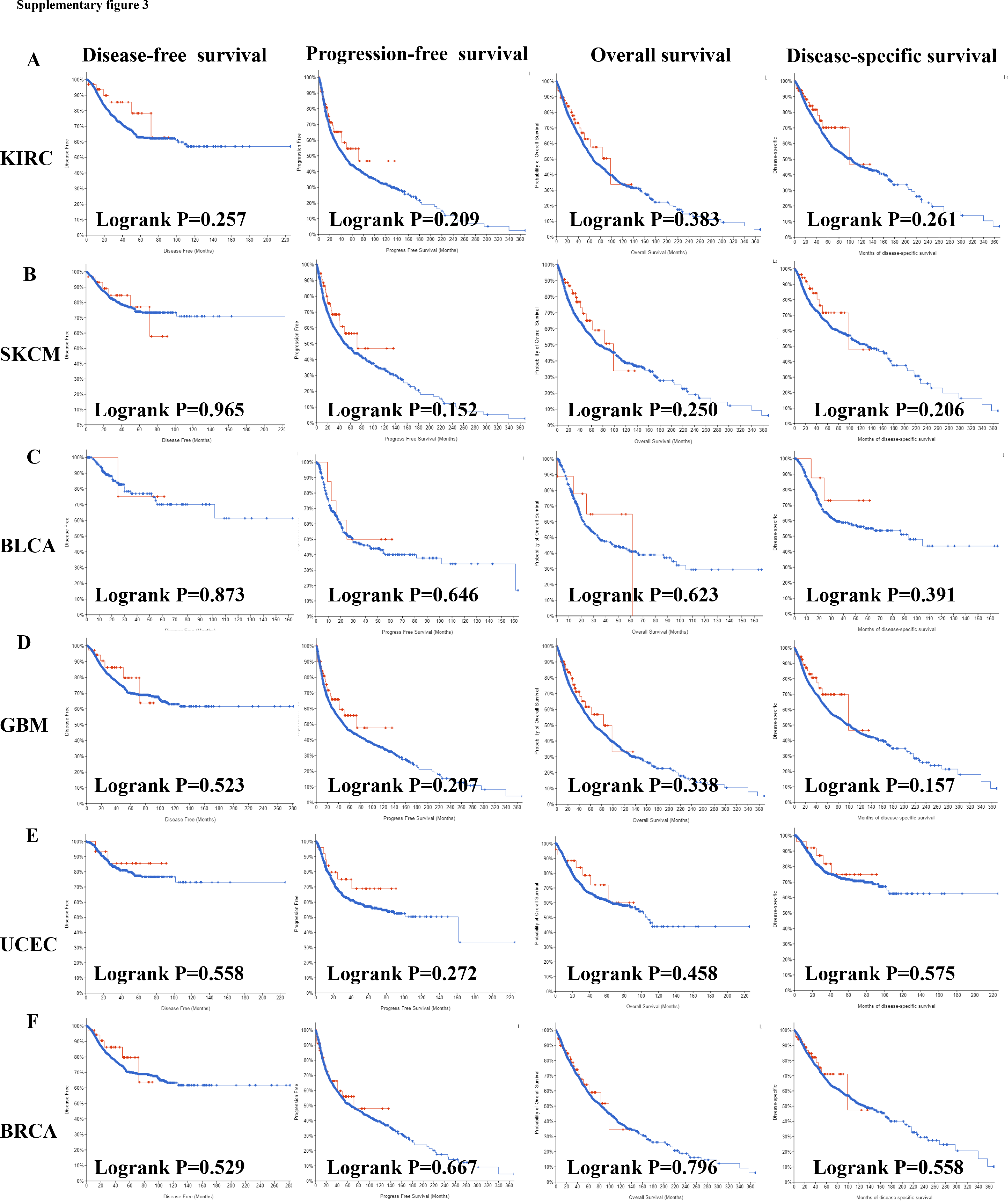

Supplement: Supplementary file 2 [file Image3.TIF]

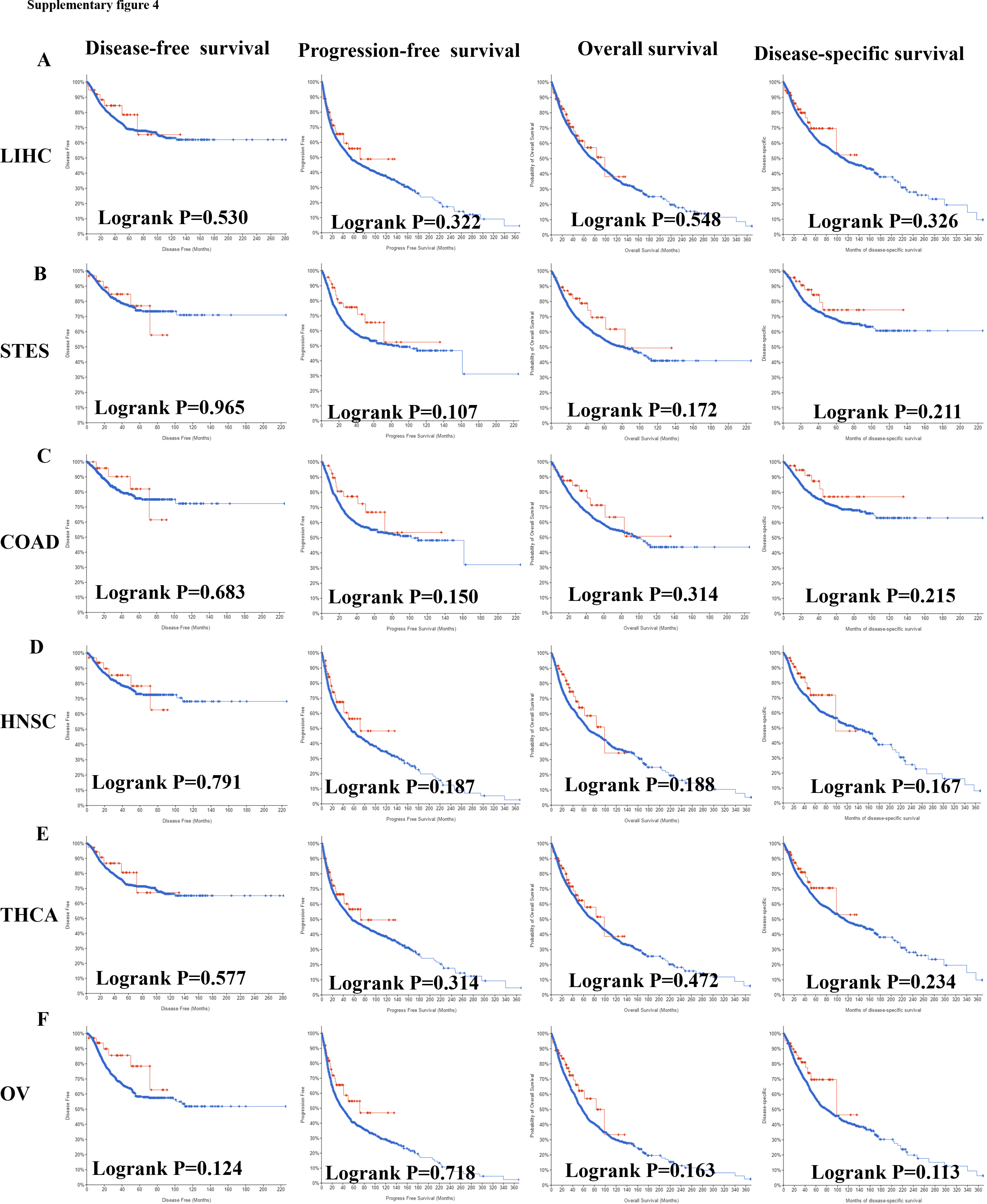

Supplement: Supplementary file 3 [file Image4.TIF]

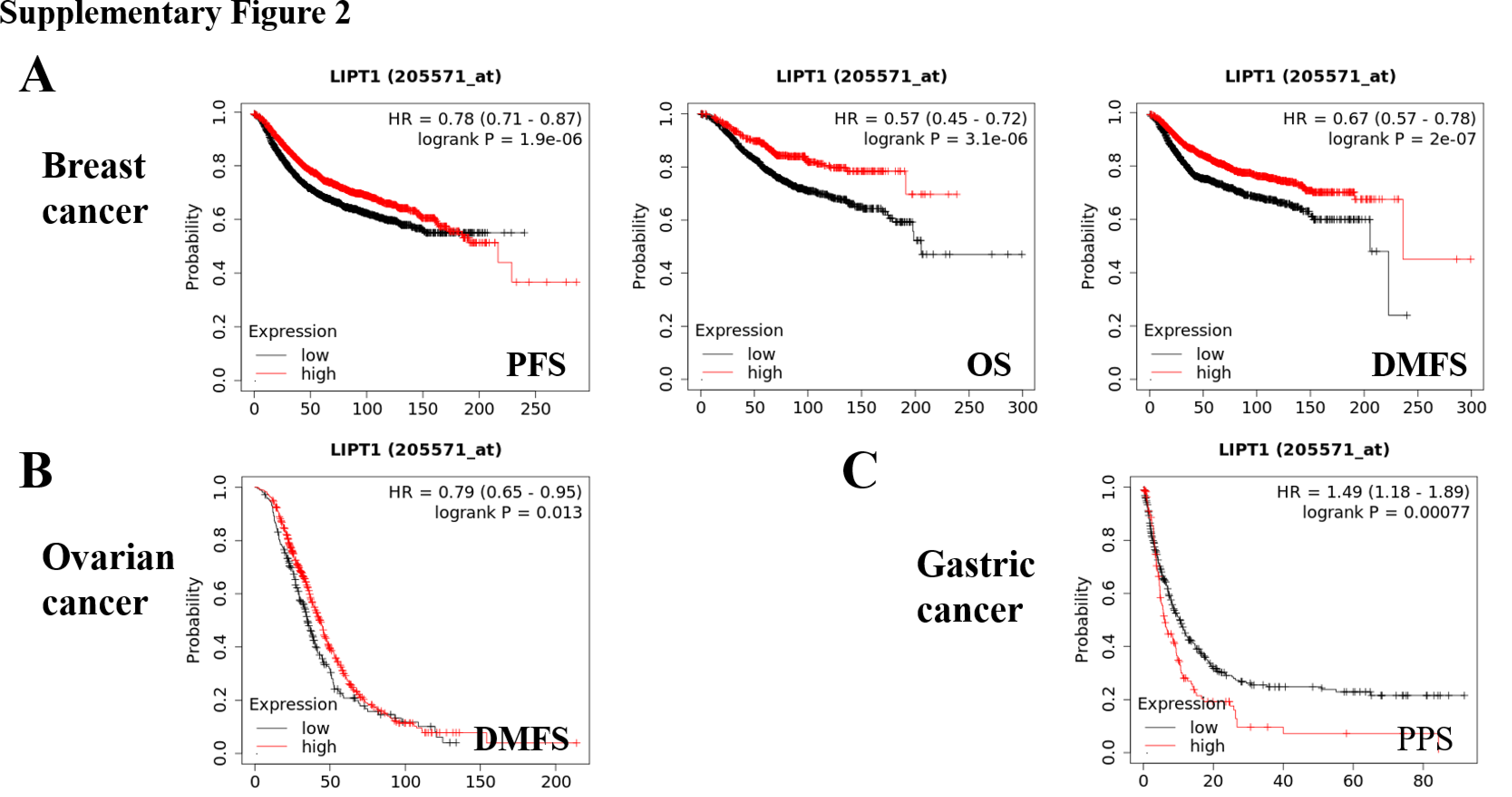

Supplement: Supplementary file 4 [file Image2.TIF]

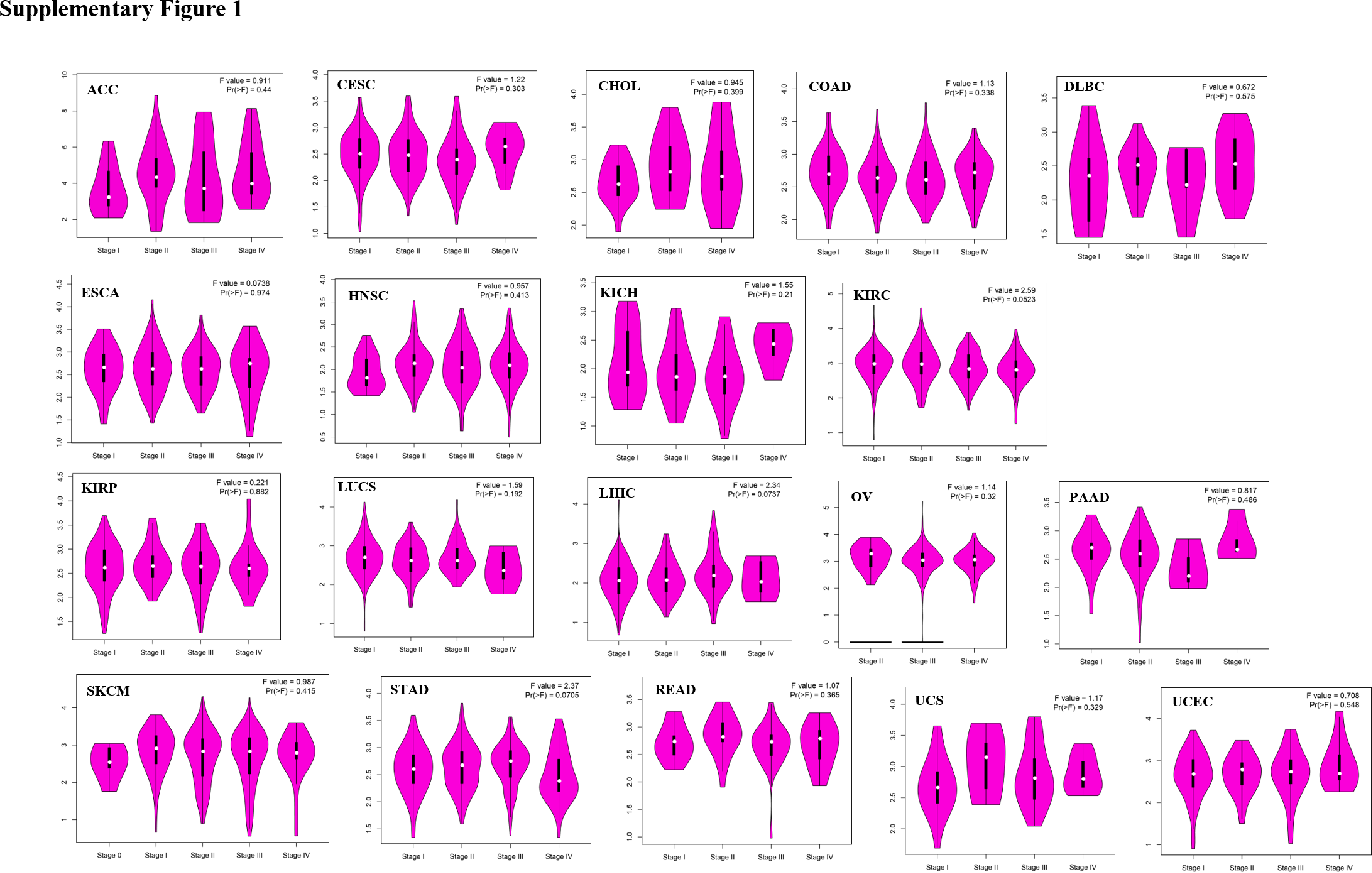

Supplement: Supplementary file 5 [file Image1.TIF]

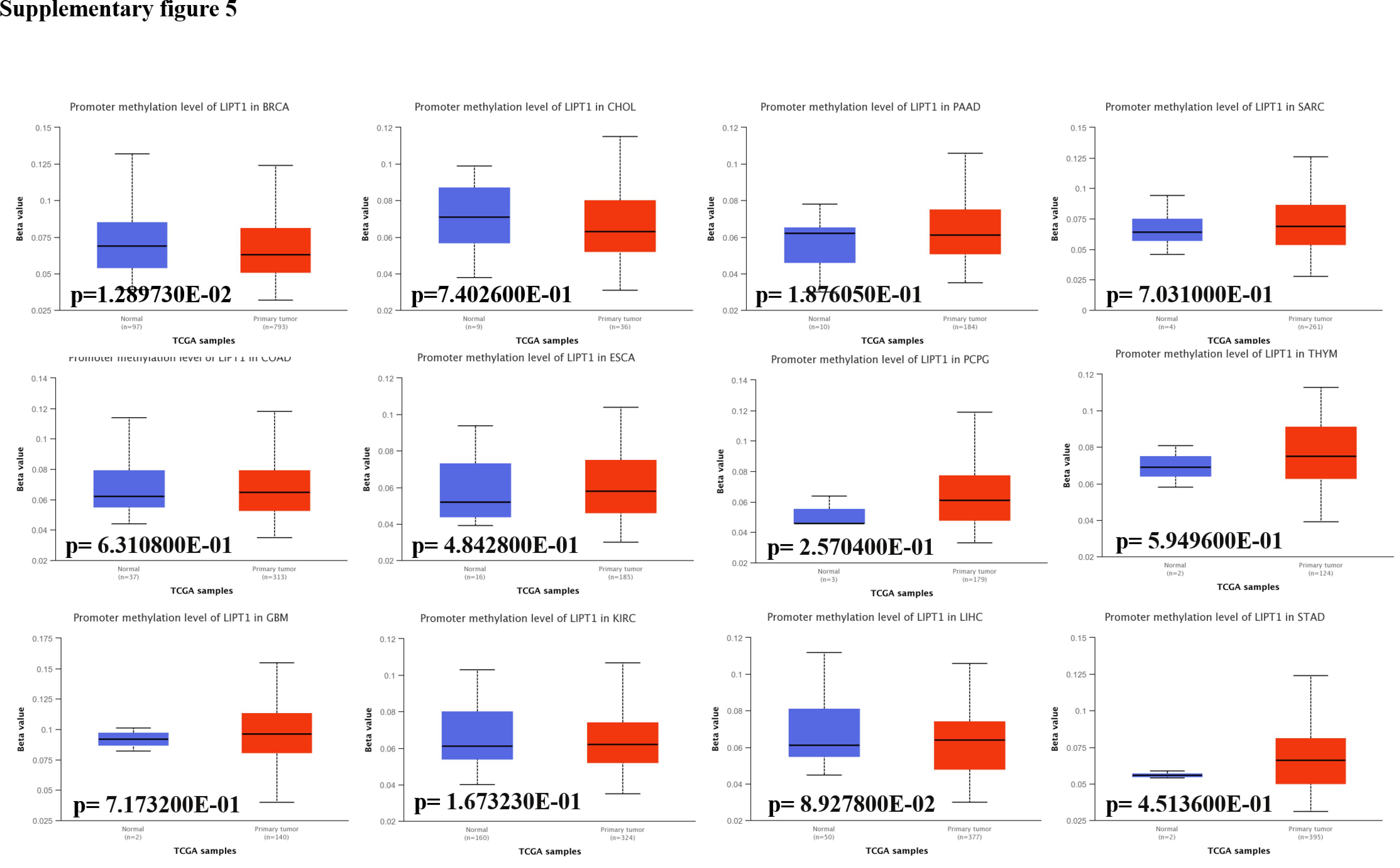

Supplement: Supplementary file 7 [file Image5.TIF]
