# Supplementary material for: A pan-cancer analysis of copper homeostasis-related gene lipoyltransferase 1: Its potential biological functions and prognosis values
Source: Front Genet. 2022 Oct 18;13:1038174. doi: 10.3389/fgene.2022.1038174 (PMC9623413; doi:10.3389/fgene.2022.1038174)
Supplement: Supplementary file 6 [file DataSheet1.PDF]

Gene Symbol	Gene ID	PCC
TSGA10	ENSG00000135951.14	0.56
ZNF14	ENSG00000105708.8	0.56
EPC2	ENSG00000135999.11	0.55
OSGEPL1	ENSG00000128694.11	0.55
CRBN	ENSG00000113851.13	0.54
WDSUB1	ENSG00000196151.10	0.54
RP11-355B11.2	ENSG00000270820.5	0.54
THAP9	ENSG00000168152.12	0.54
CCDC121	ENSG00000176714.9	0.54
WDR5B	ENSG00000196981.3	0.54
UNC50	ENSG00000115446.11	0.54
EPM2AIP1	ENSG00000178567.7	0.54
ZFP14	ENSG00000142065.13	0.53
MTERF4	ENSG00000122085.16	0.53
ZNF136	ENSG00000196646.11	0.53
ZRANB2	ENSG00000132485.12	0.52
ZNF514	ENSG00000144026.11	0.52
SP4	ENSG00000105866.13	0.52
ZNF512	ENSG00000243943.9	0.52
COA5	ENSG00000183513.8	0.52
KCTD18	ENSG00000155729.12	0.52
PMS1	ENSG00000064933.16	0.52
TMEM50B	ENSG00000142188.16	0.51
ZNF2	ENSG00000275111.4	0.51
ZSCAN26	ENSG00000197062.11	0.51
EIF3J-AS1	ENSG00000179523.4	0.51
THAP6	ENSG00000174796.12	0.51
RAD51-AS1	ENSG00000245849.6	0.51
ZBED5	ENSG00000236287.7	0.51
ORMDL1	ENSG00000128699.13	0.5
ZBTB26	ENSG00000171448.8	0.5
ZBTB14	ENSG00000198081.10	0.5
ANKRA2	ENSG00000164331.9	0.5
CIR1	ENSG00000138433.15	0.5
SENP8	ENSG00000166192.14	0.5
HKR1	ENSG00000181666.17	0.5
NIFK-AS1	ENSG00000236859.6	0.5
EPC1	ENSG00000120616.15	0.5
USP33	ENSG00000077254.14	0.5
ZNF140	ENSG00000196387.9	0.5
ZNF253	ENSG00000256771.2	0.5
LARP7	ENSG00000174720.15	0.5
GABPA	ENSG00000154727.10	0.5
ZSCAN12	ENSG00000158691.14	0.5
ZNF610	ENSG00000167554.14	0.5
ZKSCAN8	ENSG00000198315.10	0.5
KDM3B	ENSG00000120733.13	0.49
ZNF529	ENSG00000186020.12	0.49
RABL2A	ENSG00000144134.18	0.49
BRD8	ENSG00000112983.17	0.49
ZNF571	ENSG00000180479.13	0.49
CCNT2	ENSG00000082258.12	0.49
RBM43	ENSG00000184898.6	0.49
RP11-213G2.3	ENSG00000165121.10	0.49
MATR3	ENSG00000015479.17	0.49
ZNF235	ENSG00000159917.14	0.49
WDR48	ENSG00000114742.13	0.49

SENP6	ENSG00000112701.17	0.49
ARL14EP	ENSG00000152219.4	0.49
YTHDC1	ENSG00000083896.12	0.49
ZNF566	ENSG00000186017.14	0.49
SWT1	ENSG00000116668.12	0.49
ZNF182	ENSG00000147118.10	0.49
C2orf42	ENSG00000115998.7	0.48
ZNF627	ENSG00000198551.9	0.48
CRYZL1	ENSG00000205758.11	0.48
TRMT13	ENSG00000122435.9	0.48
ORC4	ENSG00000115947.13	0.48
TIGD7	ENSG00000140993.10	0.48
ZNF594	ENSG00000180626.9	0.48
ING5	ENSG00000168395.14	0.48
DNAJC27	ENSG00000115137.11	0.48
ZMYM4	ENSG00000146463.11	0.48
PCF11	ENSG00000165494.10	0.48
ZNF345	ENSG00000251247.9	0.48
RAD17	ENSG00000152942.18	0.48
ZBTB49	ENSG00000168826.15	0.48
CWC22	ENSG00000163510.13	0.48
SF3B1	ENSG00000115524.15	0.48
C7orf55-LUC7L2	ENSG00000146963.17	0.48
KIF27	ENSG00000165115.14	0.48
TIA1	ENSG00000116001.15	0.48
ANKAR	ENSG00000151687.14	0.48
ZNF879	ENSG00000234284.6	0.48
TRIM52	ENSG00000183718.5	0.48
HELQ	ENSG00000163312.10	0.48
ZNF248	ENSG00000198105.11	0.47
WDR73	ENSG00000177082.12	0.47
AC007383.3	ENSG00000227946.1	0.47
RCOR3	ENSG00000117625.13	0.47
CCDC66	ENSG00000180376.16	0.47
RP11-333E1.1	ENSG00000261879.5	0.47
ZNF280D	ENSG00000137871.19	0.47
TTC21B	ENSG00000123607.14	0.47
WDR35	ENSG00000118965.14	0.47
DCAF8	ENSG00000132716.18	0.47
ZNF542P	ENSG00000240225.10	0.47
SPG11	ENSG00000104133.14	0.47
ZNF709	ENSG00000242852.6	0.47
POLI	ENSG00000101751.10	0.47
